# Self-reported psychological distress during the COVID-19 outbreak in Nepal: findings from an online survey

**DOI:** 10.1186/s40359-020-00497-z

**Published:** 2020-12-09

**Authors:** Kamal Gautam, Ramesh P. Adhikari, Aman Sen Gupta, Rajan Kumar Shrestha, Pitambar Koirala, Suraj Koirala

**Affiliations:** 1 Transcultural Psychosocial Organization Nepal (TPO Nepal), Baluwatar, Kathmandu, Nepal; 2 Suaahara II, Helen Keller International Nepal, Lalitpur, Nepal; 3 Padma Kanya Multiple Campus, Tribhuvan University, Kathmandu, Nepal

**Keywords:** Coronavirus, Nepal, Perceived psychological distress, Social media, Survey

## Abstract

**Background:**

A lower respiratory tract infection caused by novel coronavirus termed as Corona Virus Disease (COVID-19) was first identified in China and subsequently took the form of pandemic. Studies on disease outbreak in the past and recent COVID-19 outbreak have demonstrated increased psychological distress and adverse impacts on mental health and psychological wellbeing of people. However, the impact of COVID-19 on psychological wellbeing of people in Nepal hasn't been studied adequately. So, this paper aims to report the findings from a social media survey on psychological impacts of COVID-19 in Nepal.

**Methods:**

Data were collected through social media from 2082 Nepalese respondents between 23rd April, 2020 and 3rd May, 2020. A total of 2014 respondents who were currently residing in Nepal were included in the analysis.

**Results:**

The study suggested that half of the respondents suffered from at least one symptom of psychological distress whereas 32% suffered from two or more symptoms of psychological distress such as restlessness, fearfulness, anxiety and worry and sadness in the past 2 weeks preceding the survey date. The findings further suggested that respondents having lower family income, residing in rented room, and participants from province 2 were more likely to suffer from both single and multiple symptoms of psychological distress.

**Conclusion:**

The study has shown high prevalence of psychological distress amongst the Nepalese respondents following COVID-19 outbreak. Appropriate mental health and psychosocial support response needs to be instituted to adequately respond to psychological impacts of the epidemic.

## Background

A febrile respiratory syndrome due to a pneumonia caused by a new coronavirus was first identified in China in December 2019 [1]. Subsequently, a cluster of patients with pneumonia of unknown cause was linked to a local Huanan South China Seafood Market in Wuhan, Hubei Province, China [2]. The World Health Organization (WHO) used the term 2019 novel coronavirus to refer to the virus that affected the lower respiratory tract of patients with pneumonia in Wuhan, China on 29 December 2019 [1]. The World Health Organization announced that the official name of this virus is coronavirus disease (COVID-19). The current reference name for the virus is severe acute respiratory syndrome coronavirus 2 (SARS-CoV-2).

COVID-19 has now been declared as a Public Health Emergency of International Concern by the WHO. COVID-19 has infected 4,986,332 (https://www.worldometers.info/coronavirus/) (Last updated on May 20, 2020, 02:21 GMT) people. The number of diagnosed cases of COVID-19 has been increasing rapidly daily and the number of deaths from infection has reached to 324,910 (Last updated: May 20, 2020, 02:21 GMT) globally.

Studies conducted in China following COVID-19 epidemic have demonstrated increasing psychological problems including anxiety, depression and stress in the general population alongside the observed increase in the number of diagnosed cases and deaths from COVID-19 [3, 4]. Studies on populations quarantined following disease outbreak have demonstrated increased psychological symptoms [5]; stress [6]; low mood, irritability and insomnia [7]; post-traumatic stress symptoms [8], anger [9] and emotional exhaustion [10].

A total of 402 COVID-19 positive cases have been detected in Nepal including 2 deaths till 20^th^ May, 2020 (https://www.worldometers.info/coronavirus/) (Last updated on May 20, 2020, 02:21 GMT) and WHO has categorized Nepal as a country at-high-risk. Nepal could have been categorized into high-risk due to multiple factors as- increasing number of Nepalese students going to China for higher education, influx of Chinese workers into various development projects in Nepal, large number of people involved in informal sectors, porous border with India and increasing mobility in the aftermath of COVID-19 outbreak [11–13]. The Government of Nepal declared a nationwide lockdown with effect from 24th March, 2020 [14]. People have been compelled to stay at home in quarantine and few hundreds in isolation following diagnosis. A review of the international literature on psychological impact of quarantine was conducted by Brooks et al. using 3 electronic databases and included 24 out of 3166 papers initially screened. This review of studies done outside Nepal across 10 countries following Severe Acute Respiratory Syndrome (SARS), Ebola, Middle East Respiratory Syndrome (MERS), H1N1 influenza and equine influenza outbreak identified a number of stressors over quarantine period. These include duration of quarantine, fears of infection, frustration and boredom, inadequate supplies, inadequate information, finances and stigma [15].

A number of studies have been conducted on the prevalence of mental disorders in the Nepalese population prior to the epidemic of COVID-19 in the country. A pilot study was conducted as a part of the National Mental Health Survey using MINI International Neuropsychiatric Interview (MINI) tools for adolescents and adults version 7.0.2 in between 2017–2018. It included 1647 participants (1371 adults above 17 years and 276 adolescents aged 13–17 years) from three ecological regions of the country. The study showed that the current prevalence of any form of mental disorders was 12.9%. Similarly, current major depressive disorder was found among 0.7% adolescents and 3.4% adults [16]. A nationwide cross-sectional study conducted among representative adult sample in Nepal in 2013 using Hospital Anxiety and Depression Scale (HADS) demonstrated the age- and gender-adjusted point prevalences of anxiety and depression as 16.2% and 4.1% respectively [17]. A systematic review of studies on impact of COVID-19 pandemic on mental health in the general population in various countries including Nepal showed relatively high rates of symptoms of anxiety, depression, post-traumatic stress disorder, psychological distress and stress [18]. Under this pretext, the psychosocial impacts on Nepalese population need to be studied. However, the impact of COVID-19 on psychosocial wellbeing in Nepal hasn't been studied adequately. So, we aim to explore the impact of COVID-19 on psychological wellbeing of people in Nepal using the social media survey. Hence, assessing the different nature of psychological distress and factors associated with them will provide insights into the development and implementation of effective community based psychosocial programs in the community.

## Method

This paper uses data from the COVID-19 social media survey 2020 (Additional file 1: Appendix A-Consent and Survey Questionnaire). The main objective of this survey was to understand people's perceptions of COVID-19 and its impact on psychological wellbeing. This survey included information on a wide range of symptoms of psychological distress, perception on COVID-19 along with socio-economic and demographic characteristics. Before starting the survey, a short questionnaire was developed in discussion with different stakeholders such as health workers, researcher and academicians. After finalizing the survey questionnaire, a program was developed on Microsoft forms. The social media survey was done through Facebook which is the widely used social media in Nepal with an extensive reach to a large number of respondents. The survey questionnaire developed on Microsoft forms with an online link was shared through Facebook page of research team at 4 PM on 23rd April, 2020. The link was shared among the network of research team comprising of friends, health workers, civil society organizations, counsellors and other representatives from all provinces in Nepal to ensure representation from across the country. Respondents were requested to attend the survey and to share the online link in their social network. Additionally, they were also requested not to participate more than once in the survey. The survey ended on 3rd May, 2020. The data were accessible to the research team via online link. To anonymize the respondents during the survey, the system didn't require respondent's name or email address. The participation of respondents was voluntary, and respondents had the right to decline from the survey after going through objectives. During this period, 2082 Nepalese respondents within and outside the country participated in the survey. Out of them, 2014 respondents were currently residing in Nepal and were considered for this analysis.

Four different symptoms of psychological distress experienced by respondents in the last 2 weeks preceding the survey were analyzed to assess the perceived psychological distress. The symptoms of psychological distress preceding the survey were (a) felt restless in recent circumstance (b) became fearful on thinking about recent circumstance; (c) felt anxious and worried; and (d) felt sad. The four items fearfulness, anxiety and worry, restlessness and sadness used in the survey represent most common psychological constructs of anxiety and depression. These were chosen based on the review of literature and consensus amongst the investigators. The responses to each question ranged from never experienced, experienced sometimes, most of the times and always. Based on the responses of each four items, a composite term moderate/severe psychological distress (experience of either most of the times or always in any of the four situations) was generated and treated as a dependent variable. In addition to this experience of multiple symptoms of psychological distress, only one symptom or more than one symptom were generated and treated as dependent variables.

Some potential socioeconomic and demographic confounders such as age; sex; marital status (married and unmarried); children under 2 years and pregnancy in the household; types of current residence (rented house and own house); current occupation (agriculture/labour/household work, service, business, health worker and student); family income (less than Rs 10,000, Rs 10,000–20,000, Rs 20,001–50,000 and above Rs 50,000); province (province 1, province 2, Bagmati province excluding Kathmandu valley; only Kathmandu Valley; Gandaki province, province 5, Karnali province and Sudurpashchim province) and ecological region (mountain/hill and terai) were in the adjusted models. The occupational categories in the study have been defined respectively. Agriculture has been defined as any farming related activity. Labour has been defined as any productive work done on a wage basis. Similarly, household work has been defined as routine care activities within a family. Service has been defined herein as any government or non-government job excluding health care. Health worker has been defined as any government or non-government health care professional ranging from non-specialist to specialist. Business has been defined as any form of entrepreneurship. Student has been defined as any individual pursuing his/her studies. Though health care would generally come under service, health workers have been identified as a separate category for our study. Health workers in Nepal have been working on frontline to provide various preventive, promotive and treatment services to individuals at-risk and those infected with COVID-19. So, they are presumed to have additional responsibilities and to be under higher levels of stress following the COVID-19 epidemic in Nepal. This is in contrast to other categories within “service”. So, the “health workers” have been treated as a category distinct to “service” in the study and then analyzed separately. To identify the possible predictors of psychological distress, logistic regression models were used. All available confounders were adjusted in the final model. All the analysis was performed in STATA 14.

## Results

### Characteristics of the study population

Of the total 2014 participants, around 70% were male and majority were residing in their own house (63.4%). Slightly more than one third (35.9%) of the respondent’s current occupation was service and more than one third (37.4%) of the respondent’s average monthly income ranged from Rs 20,001 to 50,000. Around two-third of the respondents were from hilly and mountainous regions (Table 1).

**Table 1:** Sociodemographic characteristics of study participants

Background characteristics	%	N
Sex		
Male	69.5	1400
Female	30.5	614
Age groups		
Less than 25 years	13.2	266
25–30 years	28.4	572
31–35 years	23.6	476
36–40 years	15.5	312
Above 40 years	19.3	388
Marital status		
Unmarried	30.2	608
Married	69.8	1406
Children under 2 years and pregnancy in the household		
Pregnancy only	5.9	119
Children under 2 years only	17.4	350
Pregnancy and children under 2 years	7.0	140
Neither	69.8	1405
Current residence		
Own house	63.4	1277
Rented room/house	36.6	737
Occupation		
Agriculture/labour/household work	9.6	193
Service	35.9	724
Business	8.1	164
Health worker	20.2	407
Student	13.8	278
Other	12.4	248
Family monthly income		
Less than 10,000	12.2	245
10,000–20,000	23.6	475
20,001–50,000	37.4	753
Above 50,000	26.9	541
Province		
Province 1	8.8	178
Province 2	7.5	151
Bagmati Province (only capital city)	37.3	752
Bagmati Province (excluding capital city)	8.4	215
Gandaki Province	10.7	207
Province 5	10.3	159
Karnali Province	7.9	182
Sudurpashchim Province	9.0	170
Ecological zone		
Hill/mountain	66.6	1341
Terai	33.4	673
Total	100.0	2014

### Perceived psychological distress

Table 2 presents the perceived psychological distress of the respondents due to COVID-19 by background characteristics. Around one fifth of the participants reported three different symptoms of psychological distress such as fearfulness, anxiety and worry, and sadness. More than one third (35%) of the respondents reported anxiety and worry in the last 2 weeks. Background characteristics indicated that more female than male; respondents having children under 2 years and pregnancy in the household; respondents having agriculture/labour/household work as occupation compared with other occupations; and respondents having lower family income had suffered more from the perceived psychological distress.

**Table 2:** Perceived psychological distress due to COVID-19

Background characteristics	Moderate/severe psychological distress				N
	Restlessness (%)	Fearfulness (%)	Anxiety and worry (%)	Sadness (%)	
Sex					
Male	17.7	16.9	34.6	27.7	1400
Female	20.0	21.3	37.3	31.4	614
Age group					
Less than 25 years	20.7	24.4	39.5	35.0	266
25–30 years	18.2	16.1	35.5	30.1	572
31–35 years	17.6	18.1	35.5	28.2	476
36–40 years	21.8	20.5	40.7	30.4	312
Above 40 years	15.5	15.5	28.1	22.4	388
Marital status					
Unmarried	20.4	18.3	37.5	32.6	608
Married	17.6	18.2	34.5	27.2	1406
Children under 2 years and pregnancy in the household					
Pregnancy only	18.5	20.2	35.3	26.9	119
Children under 2 years only	20.0	17.1	39.7	30.0	350
Pregnancy and children under 2 years	21.4	19.3	37.9	33.6	140
Neither	17.7	18.2	34.1	28.3	1405
Current residence					
Own house	16.7	17.4	31.3	26.3	1277
Rented room/house	21.4	19.7	42.6	33.2	737
Occupation					
Agriculture/labour/household work	32.6	26.4	54.4	40.4	193
Service	13.8	15.9	30.9	25.0	724
Business	24.4	20.7	31.1	31.7	164
Health worker	13.3	14.5	28.0	22.4	407
Student	23.4	22.3	41.7	38.1	278
Other	19.8	18.5	41.5	29.4	248
Family monthly income					
Less than 10,000	32.7	29.0	54.7	44.1	245
10,000–20,000	22.1	21.5	48.6	37.3	475
20,001–50,000	14.1	15.7	30.3	26.7	753
Above 50,000	14.8	14.0	22.2	17.6	541
Province					
Province 1	19.1	18.0	38.8	30.9	178
Province 2	27.2	32.5	51.7	41.7	151
Bagmati Province (only Capital city)	17.7	16.6	35.5	27.4	752
Bagmati Province (excluding Capital city)	14.0	12.1	31.8	24.7	215
Gandaki Province	17.4	20.3	32.6	28.0	207
Province 5	20.8	22.0	26.6	31.4	159
Karnali Province	23.1	23.6	35.2	34.1	182
Sudurpashchim Province	12.9	8.8	35.2	20.0	170
Ecological zone					
Hill/mountain	12.6	16.9	35.0	28.1	1341
Terai	20.1	21.0	36.3	30.3	673
Total	18.4	18.2	35.4	28.8	2014

Only the percentage of individuals having distress has been included in the table

In the last 2 weeks, around half of the respondents suffered from at least one symptom of psychological distress whereas around one third (32.2%) suffered from two or more symptoms of psychological distress such as restlessness, fearfulness, anxiety and worry, and sadness. Disaggregated analysis suggested that females; respondents having children under 2 years and pregnancy in the household; and respondents having lower family income had suffered more from either at least one or two or more symptoms of psychological distress (Table 3).

**Table 3:** Multiple symptoms of psychological distress during COVID-19

Background characteristics	At least one symptom (restlessness/fearfulness/anxiety and worry/sadness) (%)	N	Two or more symptoms (restlessness/fearfulness/anxiety and worry/sadness) (%)	N
Sex				
Male	48.2	1400	30.7	1400
Female	53.7	614	35.5	614
Age group				
Less than 25 years	58.6	266	39.8	266
25–30 years	51.6	572	33.6	572
31–35 years	48.1	476	30.9	476
36–40 years	51.9	312	34.9	312
Above 40 years	42.0	388	24.2	388
Marital status				
Unmarried	53.6	608	36.5	608
Married	48.3	1406	30.3	1406
Children under 2 years and pregnancy in the household				
Pregnancy only	54.0	119	32.0	119
Children under 2 years only	52.0	350	35.0	350
Pregnancy and children under 2 years	48.0	140	31.0	140
Neither	49.0	1405	31.0	1405
Current residence				
Own house	46.0	1277	28.9	1277
Rented room/house	56.7	737	37.9	737
Occupation				
Agriculture/labour/household work	68.9	193	49.7	193
Service	45.2	724	26.5	724
Business	48.2	164	32.9	164
Health worker	39.1	407	22.6	407
Student	60.8	278	43.5	278
Other	55.6	248	37.5	248
Family monthly income				
Less than 10,000	71.8	245	57.1	245
10,000–20,000	62.5	475	42.7	475
20,001–50,000	45.2	753	25.9	753
Above 50,000	35.5	541	20.3	541
Province				
Province 1	53.4	178	35.4	178
Province 2	66.2	151	48.3	151
Bagmati Province (only Capital city)	48.8	752	31.6	752
Bagmati Province (excluding Capital city)	40.6	170	22.4	170
Gandaki Province	46.5	215	24.2	215
Province 5	44.9	207	28.0	207
Karnali Province	53.5	159	37.1	159
Sudurpashchim Province	52.7	182	36.8	182
Ecological zone				
Hill/mountain	49.2	1341	30.8	1341
Terai	51.3	673	34.8	673
Total	49.9	2014	32.2	2014

Only the percentage of individuals having distress has been included in the table

### Factors associated with psychological distress

Table 4 provides the results from analyses of adjusted and unadjusted associations of different socio-economic characteristics with the experience of single or multiple symptoms of psychological distress. Adjusted logistic regression model indicated that respondents residing in rented room increased the odds of suffering from both single (Adjusted OR 1.56, CI: 1.28–1.91) and multiple symptoms of psychological distress (Adjusted OR 1.54, CI: 1.24–1.90). Similarly, people residing in province 2 were more likely to suffer from both single (Adjusted OR 1.80, CI: 1.12–2.89) and multiple (Adjusted OR 1.79, CI: 1.12–2.87) symptoms of psychological distress whereas the participants residing in Bagmati province (excluding capital city) were less likely to suffer from both single (Adjusted OR 0.62, CI: 0.39–0.98) and multiple symptoms of psychological distress (Adjusted OR 0.57, CI: 0.34–0.94). Increase in the monthly income of the households reduced the odds of suffering from either single (Adjusted OR 0.29, CI: 0.20–0.41) or multiple symptoms of psychological distress (Adjusted OR 0.25, CI: 0.18–0.36).

**Table 4:** Factors associated with psychological distress during COVID-19

Background characteristics	At least one symptom		Two or more symptoms	
	Unadjusted	Adjusted^a^	Unadjusted	Adjusted^a^
	OR (95% CI)	OR (95% CI)	OR (95% CI)	OR (95% CI)
Sex				
Male	0.79* (0.66–0.96)	0.80* (0.65–0.99)	0.81* (0.67–0.99)	0.81 (0.65–1.02)
Age	0.98*** (0.97–0.99)	0.99 (0.98–1.01)	0.98*** (0.97–0.99)	1.00 (0.98–1.01)
Marital status				
Unmarried				
Married	0.81* (0.67–0.98)	1.05 (0.81–1.36)	0.76** (0.62–0.92)	0.96 (0.73–1.26)
Children under 2 years and pregnancy in the household				
Neither				
Pregnancy only	0.95 (0.65–1.38)	0.88 (0.59–1.32)	1.06 (0.71–1.58)	0.99 (0.64–1.52)
Children under 2 years only	1.11 (0.88–1.40)	1.00 (0.77–1.29)	1.18 (0.92–1.51)	1.07 (0.81–1.40)
Pregnancy and children under 2 years	1.23 (0.87–1.74)	1.18 (0.81–1.71)	1.00 (0.69–1.45)	0.92 (0.62–1.38)
Current residence				
Own house				
Rented room/house	1.54*** (1.28–1.85)	1.56*** (1.28–1.91)	1.50*** (1.24–1.82)	1.54*** (1.24–1.90)
Occupation				
Agriculture/labour/household work				
Service	0.37*** (0.26–0.52)	0.58** (0.40–0.85)	0.36*** (0.26–0.51)	0.61** (0.42–0.87)
Business	0.42*** (0.27–0.65)	0.68 (0.43–1.08)	0.50*** (0.32–0.76)	0.89 (0.56–1.42)
Health worker	0.29*** (0.20–0.42)	0.42*** (0.28–0.62)	0.30*** (0.20–0.43)	0.45*** (0.30–0.68)
Student	0.70 (0.47–1.03)	0.74 (0.48–1.14)	0.78 (0.54–1.13)	0.80 (0.53–1.23)
Other	0.57** (0.38–0.84)	0.83 (0.54–1.26)	0.61** (0.41–0.89)	0.90** (0.59–1.36)
Family monthly income				
Less than 10,000				
10,000–20,000	0.65** (0.47–0.91)	0.72 (0.51–1.02)	0.56*** (0.41–0.76)	0.60** (0.43–0.83)
20,001–50,000	0.32*** (0.24–0.44)	0.42*** (0.30–0.59)	0.26*** (0.19–0.35)	0.34*** (0.24–0.47)
Above 50,000	0.22*** (0.16–0.30)	0.29*** (0.20–0.41)	0.19*** (0.14–0.27)	0.25*** (0.18–0.36)
Province				
Province 1				
Province 2	1.71** (1.09–2.68)	1.80* (1.12–2.89)	1.71* (1.10–2.66)	1.79* (1.12–2.87)
Bagmati Province (only Capital city)	0.83 (0.60–1.16)	0.88 (0.59–1.31)	0.85 (0.60–1.19)	0.97 (0.64–1.49)
Bagmati Province (excluding Capital city)	0.59* (0.39–0.91)	0.62* (0.39–0.98)	0.53** (0.33–0.84)	0.57* (0.34–0.94)
Gandaki Province	0.76 (0.51–1.13)	0.83 (0.53–1.30)	0.58* (0.38–0.90)	0.68 (0.41–0.11)
Province 5	0.71 (0.48–1.07)	0.80 (0.53–1.22)	0.71 (0.46–1.09)	0.81 (0.51–1.28)
Karnali Province	1.00 (0.65–1.54)	1.03 (0.63–1.68)	1.08 (0.69–1.68)	1.21 (0.72–1.02)
Sudurpashchim Province	0.98 (0.64–1.48)	1.02 (0.66–1.57)	1.06 (0.69–1.64)	1.13 (0.72–1.79)
Ecological zone				
Hill/mountain				
Terai	1.08 (0.90–1.31)	0.99 (0.75–1.32)	1.19 (0.98–1.45)	1.12 (0.83–1.52)

OR, odd ratio; CI, confidence interval

*p < 0.05; **p < 0.01; ***p < 0.001

^a^adjusted for sex, marital status, children under 2 years and pregnancy in the household, current residence, occupation and family monthly income

## Discussion

This social media survey has generated evidence related to the impact of COVID-19 on psychological wellbeing of people in Nepal. There are limited academic studies which have studied the impact of COVD-19 pandemic on the mental health and psychological wellbeing of people [19]. To the best of our knowledge, this study is the first large scale survey of psychological distress in the Nepalese population following the COVID-19 outbreak. Due to the way the survey was constructed, all participants completed all items, which was a further strength of the study.

The study suggests that half of the respondents suffered from at least one symptom of psychological distress whereas 32% suffered from two or more symptoms of psychological distress such as restlessness, fearfulness, anxiety and worry, and sadness in the past 2 weeks preceding the survey date. The findings further suggest that respondents having lower family income, residing in rented room, and participants from province 2 were more likely to suffer from both single and multiple symptoms of psychological distress. On the other hand, health workers, those currently involved in regular service, and the respondents from the Bagmati province (excluding capital city) were less likely to suffer from either single or multiple symptoms of psychological distress.

The outbreak of COVID-19 has raised concerns about the potential for a widespread increase in mental health issues [20–22]. People could experience fear of death, fear of getting oneself or their family infected, anxiety, anger, depressive symptoms, and other mental health concerns during this pandemic outbreak [23]. COVID-19 pandemic outbreak could have negative impact on psychological and mental health of people, for instance psychological distress, mental health issues, grief, shame, helplessness, hopelessness, posttraumatic symptoms, substance abuse, panic attacks, stress, anxiety, depression, loneliness, ambivalence, fear, anger, stigma and worry towards socioeconomic status [24]. Similar findings have been replicated in our study. Our study has also revealed increased prevalence of psychological distress such as restlessness, fearfulness, anxiety and worry, and sadness among the participants following COVID-19 outbreak. The prevalence of symptoms of psychological distress seem to have increased following COVID-19 outbreak and lockdown when compared to data available from existing literature prior to the epidemic. A nation-wide cross sectional study conducted among Nepalese adults between 18–65 years showed the age- and gender-adjusted prevalence of anxiety, depression and co-morbid anxiety and depression as 16.1, 4.2 and 5.9% respectively [17]. However, the data is comparable to other humanitarian settings as major earthquake of 2015. A representative cluster sample survey conducted by TPO Nepal four months post-earthquake in three affected districts demonstrated elevated rates for depression (34.3%) and anxiety (33.8%) which were higher than prior epidemiologic surveys conducted using similar measures [25].

Our study has demonstrated that the prevalence of psychological distress is higher among households with low income and those residing in rented room/house. Poverty and low socioeconomic status are identified factors associated with poor mental health and increased psychological distress [26]. A systematic review of the epidemiological literature on common mental disorders and poverty in low-and middle-income countries found that of the 115 studies reviewed, over 70% reported positive associations between a variety of poverty measures and common mental disorders [27]. The finding of our survey is consistent with the findings from systematic review.

Similarly, the prevalence of psychological distress has been higher among female than male. This is consistent with the finding from previous studies. Epidemiological surveys have consistently documented significantly higher rates of internalizing disorders as anxiety and mood disorders among women than men [28–31]. Our study focused on symptoms of psychological distress characteristic of internalizing disorders as restlessness, fearfulness, anxiety and worry, and sadness. Thus, our finding on greater prevalence of psychological distress is similar to the findings from the epidemiological surveys on gender differences on internalizing disorders.

Our study has demonstrated lower prevalence of psychological distress amongst the health workers and those having service as an occupation. Service category includes banking, education, communication, engineering and construction works, etc. Similarly, health workers include Auxiliary Health Workers (AHWs), Health Assistants (HAs), Auxiliary Nurse Midwives (ANMs), Staff Nurses (SNs) and medical doctors. Poor quality employment, such as employment with no or short-term contracts, and jobs with low reward and control at work, have significant harmful impacts on mental health [32]. Conversely, job security and a sense of control at work are protective of good mental health [33, 34]. Health workers and respondents enrolled in service have relatively better job securities and sense of control than other occupations as labour including students who are dependent. This might have contributed to lower prevalence of psychological distress among health workers and individuals enrolled in service. Contrastingly, studies from China on mental health impacts of COVID-19 on health professionals demonstrated increasing psychological problems including anxiety, depression and stress with increase in the number of diagnosed cases and deaths from the disease [3, 4]. This might be due to the fact that Nepal is at the beginning of epidemic with limited number of cases diagnosed with COVID-19 and health system being far less overwhelmed than China where thousands of cases had been detected positive.

Additionally, our study has demonstrated higher prevalence of psychological distress in respondents from Province 2. A study entitled "Provincial Comparison of Development Status in Nepal: An Analysis of Human Development Trend for 1996 to 2026" demonstrated that the province had lower HDI, lowest literacy rate, lower per capita income and lowest increment in HDI between 1996 to 2011 [35]. Education and HDI are significant social determinants of mental health. Similarly, gender disparities, poor women empowerment, gender discrimination and limited access of women to income generating activities are other associated factors contributing to poor socioeconomic status. Since these social determinants are adverse in Province 2, it is pertinent that respondents from this province reported having higher prevalence of psychological distress over past 2 weeks of the study period. Province 2 lies adjacent to border of India and has a high influx of migrant workers and Indian citizens to Nepal for various purposes [36]. This puts the province into ongoing risk of COVID-19 transmission. Additionally, the status of quarantine and isolation centres in the province are not satisfactory. A number of newspaper articles and reports from the province reflect lack of access to basic health and sanitation facilities; lack of personal protective gears; poor security and lack of hygienic food in the quarantine and isolation centres. Similarly, the health facilities and health indicators in the province are already in a poor state [37]. All these factors might have accounted for increase in odds of distress in respondents from Province 2.

### Limitations

The study has several limitations. First, the survey was done online and included people using social media within the network of investigators. Thus, the survey might not represent a larger population outside the social network of investigators and not using social media. Secondly, the study did not use standardized tool for anxiety and depression and has used only four constructs of psychological distress. This might not give accurate measures for anxiety and depression. The study might also have missed many other symptoms of psychological distress which the respondents could have manifested. Third, since this was a cross-sectional study, analyzing the causal relationship was not possible. Fourth, some of the possible predictors such as caste/ethnicity and education were not included in the survey questionnaire and thus their interpretation could not be done. Fifth, the study did not assess level of functioning and correlate with the presence of one or more symptoms of psychological distress in respondents. Therefore, it could not specify whether the psychological distress had resulted in impairment of socio-occupational functioning. Sixth, the investigators had no control over the respondents attending the survey more than once. This might have resulted in repetitive participation in the survey and duplication of data. Seventh, since the study did not define the focus population for the survey and the online link was recirculated in chain, the interpretation of results might be quiet tricky. Additionally, between the group differences was not hypothesized prior and could not be analyzed.

### Implications

A major adverse consequence of the COVID-19 pandemic is likely to be increased social isolation and loneliness [38] which are strongly associated with anxiety, depression, self-harm, and suicide attempts across the lifespan [39, 40]. The number of diagnosed COVID-19 cases has been increasing; the duration of nationwide lockdown is extending and economic crisis is growing. This seems to put an additional turmoil into mental health and psychological wellbeing of Nepalese population. There is likely to be a surge in the number of newly diagnosed mental disorders and the deterioration of those with existing difficulties in the aftermath of COVID-19 [41]. So, the current crisis demands creating an operational framework of mental health and psychosocial response (MHPSS) and delivering integrated MHPSS services to address these consequences.

## Conclusion

The study has shown high prevalence of psychological distress amongst the Nepalese respondents. The Government of Nepal has instituted measures to prevent and control COVID-19 epidemic in the country with regard to lockdown, social distancing, testing suspected cases and tracing contacts. These are necessary to ensure the health and safety of Nepalese population. Nevertheless, it is important to recognize and acknowledge the immediate short and longer-term mental health and psychological consequences of the current crisis [42]. The demand for mental health and psychosocial support services is likely to escalate in the near future. The health care providers should be prepared in advance to face the upcoming challenges. It is recommended that the Government of Nepal initiate appropriate steps through learning from other nations and utilizing strategies that worked well for them. This is anticipated to respond to mental health epidemic that might result in the days to come.

## Supplementary information

**Additional file 1:** Consent and Survey Questionnaire.

## Data Availability

The datasets used in this study are available from the corresponding author based on request.

## Abbreviations

CI: Confidence Interval
COVID-19: Coronavirus Disease
MERS: Middle East Respiratory syndrome
OR: Odds Ratio
ref.: Reference Category
SARS: Severe Acute Respiratory Syndrome
TPO Nepal: Transcultural Psychosocial Organization Nepal
WHO: World Health Organization
