# Supplementary material for: Self-reported psychological distress during the COVID-19 outbreak in Nepal: findings from an online survey
Source: BMC Psychol. 2020 Dec 9;8:127. doi: 10.1186/s40359-020-00497-z (PMC7724457; doi:10.1186/s40359-020-00497-z)
Supplement: Supplementary file 1 — Additional file 1: Consent and Survey Questionnaire. [file 40359_2020_497_MOESM1_ESM.docx]

**Supplementary Appendix A-Consent and Survey Questionnaire**

**Coronavirus Disease (COVID-19) Social Media Survey 2020**

Namaste! I am Dr. Kamal Gautam. I am a Psychiatrist and researcher currently working in Transcultural Psychosocial Organization Nepal (TPO Nepal). My team is conducting a social media survey to understand people's perceptions of COVID-19 and its impact on psychological wellbeing. This survey is being conducted by TPO Nepal under ethical approval from the Nepal Health Research Council (NHRC) *(Reg. No. 309/2020 P)*. You will have to respond to survey questions. The survey will take about 10-12 minutes to complete. On behalf of the research team, I would request you to participate in the survey. The participation in the survey is voluntary. Please refer to the following link for attending the survey- Bit.ly/@XYV9F5. I request you not to participate more than once.

I request you to share this link over your social network to encourage larger participation.

If you have any query about the survey, please contact-

Dr. Kamal Gautam (+977-9841949876) Email:kgautam@tponepal.org.np or

Ramesh Prasad Adhikari (+9779841740281) Email: rameshadhikaria@gmail.com

| S.N. | Questions | Answers |
| --- | --- | --- |
| 1 | Do you want to participate in this survey? | Yes----1  No----2 |
| 2 | Which province do you come from? | Province 1-----------1  Province 2------------2  Bagmati Province ----3  Gandaki Province----4  Province 5---------------5  Karnali Province ----6  Sudurpashchim Province----7  Outside Nepal----8 |
| 3 | Which geographical region do you come from? | Himalayan region------- 1  Hilly region------- 2  Terai region-------3 |
| 4 | Respondent’s Gender | Female-------1  Male-------2  Others------- 3 |
| 5 | Respondent’s Age | Age--------------- |
| 6 | Where do you reside currently? | Kathmandu valley in rented room/flat/house-------1  Kathmandu valley at own house----2  Outside Kathmandu valley in rented room/flat/house -------3  Outside Kathmandu valley at own house -------4  Others-------5 |
| 7 | What is your major occupation? | Farmer---1  Health worker---2  Security personnel ---3  Employed (besides health worker and security personnel) ---4  Business---5  Student---6  Domestic work/house wife---7  laborer---8  others--9 |
| 8 | Average monthly income of your family is? | less than 10000-------1  10000 to 20000-------2  20001 to 50000-------3  above 50000-------4 |
| 9 | Marital status | Married-------1  Unmarried-------2  Others-------3 |
| 10 | Is there a pregnant woman or a child below 2 years in your family? | has pregnant woman-------1  has child below 2 years-------2  has both-------3  do not have any-------4 |
| 11 | Though which sources have you received information on coronavirus (COVID-19)?  (choose all the options applicable/multiple choice) | Radio---1  Newspaper/magazines---2  Television---3  Health worker---4  Family member(s)---5  Neighbors/relatives---6  flyer/pamphlets---7  Social networking site (facebook)--8  Others-------9 |
| 12 | During the period of lockdown how much work did you have to do? | same as usual-------1  less tha usual-------2  more than usual--------3 |
| 13 | Did you and your family members have to face difficulty in managing food due to lockdown imposed to manage COVID-19?  (e.g. due to shortage of food or lack of appropriate food) | Not at all---0  less often/abit---1  very often/a lot---2 |
| 14 | Did you or your family members have to face severe problem/crisis due to economic hardships/financial crisis or lack of necessary resources to sustain your living? | Not at all---0  less often/a bit---1  very often/a lot---2 |
| 15 | During the period of lockdown, did you/your family members or your neighbors have to face any form of violence?  (e.g. domestic violence, sexual violence, child abuse) ( Please choose all the applicable answers/multiple choice) | No---1  incidence of child abuse---2  incidence of violence against women---3  incidence of violence over people with disability ---4  incidence of violence over elderly people---5  I do not know---6 |
| 16 | In past two weeks, how often did you feel restless due to current situation? | Never---1  Sometimes--2  Very often---3  Everyday --4 |
| 17 | In past two weeks, how often did you experiennce fearfulness due to current situation? | Never---1  Sometimes--2  Very often---3  Everyday --4 |
| 18 | In past two weeks, how often did you feel anxious and worried in the recent circumstance? | Never---1  Sometimes but didn’t affect much--2  Very often but I could cope with it--3  Very often and I could not cope with it--4 |
| 19 | In past two weeks, how often did you experience sadness? | Never---1  Sometimes but didn’t affect much--2  Very often but I could cope with it--3  Very often and I could not cope with it--4 |
| 20 | In past two weeks, how often were you anxious and worried about managing food for you and your family members? | Never---1  Sometimes but didn’t affect much--2  Very often but I could cope with it--3  Very often and I could not cope with it--4 |
| 21 | In past two weeks, how often were you anxious and worried thinking about you or your family member losing the current job or not finding a new one? | Never---1  Sometimes but didn’t affect much--2  Very often but I could cope with it--3  Very often and I could not cope with it--4 |
| 22 | What did you do to manage such stress and worries? ( please choose all the applicable answers/multiple choice) | Yoga and exercise--1  Discussing/sharing with family/friends--2  Taking advice from health worker --3  Participating in entertaining activities--4  Indulge in alcohol and other substances--5  Others--6 |
| 23 | In your opinion, what should the government do for managing COVID-19? ( Please choose all the applicable answers/multiple choice) | Continue lockdown (unless the spread is completely controlled) ---1  Lift up lockdown by maintating social distancing---2  Lockdown only where infected individuals are present---3  Increase PCR testing---4  Others--5 |
| 24 | After lifing the lockdown, what do you think will be the risk of coronavirus infection/spread? | Not much chances of spread---1  Some chances of spread---2  Rate of infection will increase rapidly---3  Can’t tell. Anything could happen---4 |
| 25 | How have the local government or other organizations in your community been distributing relief to people in need? | No one has distributed any relief ---1  Distributed to some but not to others---2  In a managed way---3  In an unmanaged/discriminatory way---4  I do not know---5 |
| 26 | Do you know about COVID-19 insurance scheme recently implemented in Nepal? | Yes---1  No---2 |
| 27 | Have you enrolled or are intending to enroll in COVID-19 insurance scheme? | Already enrolled---1  Intending to enroll---2  No---3  I do not know--4 |

Thank you for participating in the survey.
